# Acute Suppurative Thyroiditis Caused by *Gemella Morbillorum*: A Case Report

**DOI:** 10.2174/0118715303347962241018091143

**Published:** 2024-12-09

**Authors:** Alessandro Brunetti, Claudia Cipri, Assunta Sartor, Anna Maria Bergamin-Bracale, Jacopo Manso, Fabio Vescini

**Affiliations:** 1 Endocrinology Unit, Ospedale Santa Maria della Misericordia, Udine, Italy;; 2 Microbiology Unit, Ospedale Santa Maria della Misericordia, Udine, Italy;; 3 Department of Otolaryngology-Head and Neck Surgery, Ospedale Santa Maria della Misericordia, Udine, Italy

**Keywords:** Acute suppurative thyroiditis, Gemella morbillorum, thyroid infection, bacterial thyroiditis, neutrophilic leucocytosis, antibiotic therapy

## Abstract

**Background:**

Acute suppurative thyroiditis (AST) is a rare form of thyroid inflammation prevalently of bacterial origin that usually affects subjects with risk factors such as immunodeficiency, sepsis, and neck fistulas. The most prevalent pathogens associated with AST are gram-positive aerobic bacteria, followed by gram-negatives, while infections by anaerobic germs are exceptionally rare. *Gemella morbillorum* is a facultative anaerobic gram- positive bacterium that commonly populates the upper respiratory tract. Infections by *Gemella Morbillorum* have been previously documented in different regions (*i.e.,* lung, brain, bone, liver), but never in the thyroid.

**Case Presentation:**

An 18-year-old male with no previous medical history presented to the emergency department complaining of a rapidly enlarging painful neck mass in left anterior latero-cervical region progressively worsening over the last two weeks, accompanied by dysphagia and fever. Blood tests showed the presence of thyroiditis (suppressed TSH with increased free thyroxine, elevated inflammation markers and neutrophilic leucocytosis). Neck ultrasonography and CT showed a large abscess involving the left thyroid lobe and extending to the ipsilateral laterocervical region, suggesting the diagnosis of AST. Prompt antibiotic therapy was started and subsequent surgical drainage of the abscess was performed, resulting in a rapid clinical recovery and the restoration of normal thyroid function. The bacterial culture of the abscess showed exclusively the presence of *Gemella morbillorum*.

**Conclusion:**

We present the first documented case of AST caused by Gemella morbillorum in an otherwise healthy young man. Although rare, AST in immunocompetent patients is possible; prompt diagnosis and treatment of this condition are fundamental to avoid severe complications.

## 1 INTRODUCTION

Acute suppurative thyroiditis (AST) represents a rare form of thyroid inflammation, which most frequently occurs as a consequence of a bacterial infection. According to a recent review, less than 200 cases of AST have been documented in the literature [1] . The rarity of AST can be attributed to the thyroid's multiple mechanisms of protection against bacterial infection, such as the presence of a capsule, extensive blood supply, and an efficient lymphatic drainage system. Therefore, AST occurs more frequently among immunocompromised individuals or in patients presenting widespread infections or cutaneous fistulas on the neck. The typical clinical presentation of bacterial AST includes neck pain (89%), fever (82%), skin erythema (38%), and dysphagia (46%) [1]. Approximately 40% of patients also present a transient state of thyrotoxicosis due to the release of pre-formed thyroid hormone, manifesting with tachycardia, sleeplessness, diaphoresis and diarrhea [1].

The most common pathogens responsible for AST are Gram-positive aerobic bacteria, followed by gram-negative ones. Conversely, infections by anaerobic germs represent less than 10% of the reported cases and are, therefore, to be considered exceptionally rare.

Gemella morbillorum (*G. Morbillorum*) is a facultative anaerobic gram-positive bacterium that commonly populates the oropharynx, the upper respiratory tract, and the gastrointestinal system. This pathogen has prevalently been associated with endocarditis in patients with pre-existing valve damage and prosthetic valves [2, 3]. Additionally, there have been documented cases of pleural empyema [4, 5], osteo-articular infections [6, 7], brain abscess [8, 9], meningitis [10], liver abscess [11, 12], and nephritis [13], primarily involving patients with immune deficiency, implantable devices, or with other predisposing conditions.

In this report, we present a case of AST caused by *G. morbillorum* in an 18-year-old male without any known underlying medical disorder or risk factors for bacterial infections.

## 2 CASE PRESENTATION

A 18-year-old male presented to the emergency department complaining of a rapidly enlarging neck mass associated with neck pain, dysphagia, and fever, progressively worsening over the last two weeks. In addition, he reported tachycardia, significant asthenia, diaphoresis, and insomnia over the past few days. The patient had a negative personal medical history, and he was not taking any chronic medication.

**Fig. (1):**
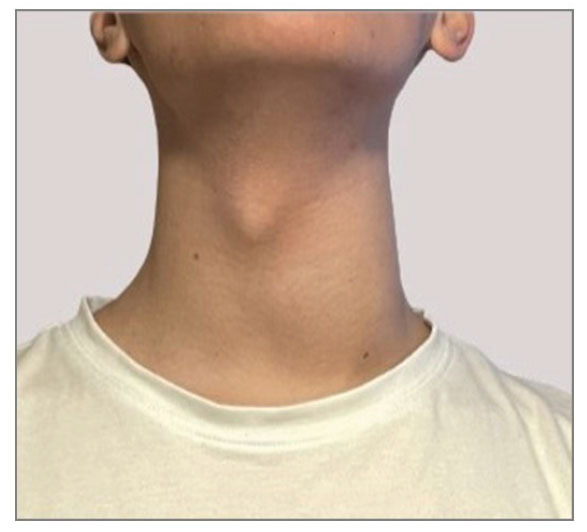
Picture of the patient at first physical examination. Swelling can be observed in correspondence of the left thyroid lobe.

**Fig. (2):**
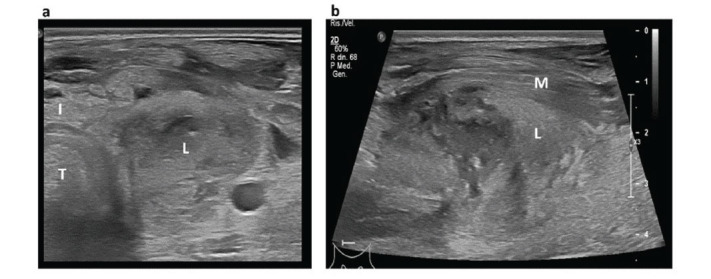
Thyroid ultrasound at time of admission, both in transversal (**a**) and longitudinal section (**b**). An inhomogeneous hypoechoic area with colliquated areas and poorly defined margins can be observed in the left thyroid lobe, which extends to the anterior neck muscles. T=trachea, I: thyroid isthmus, L=left thyroid lobe. M=muscle.

**Fig. (3):**
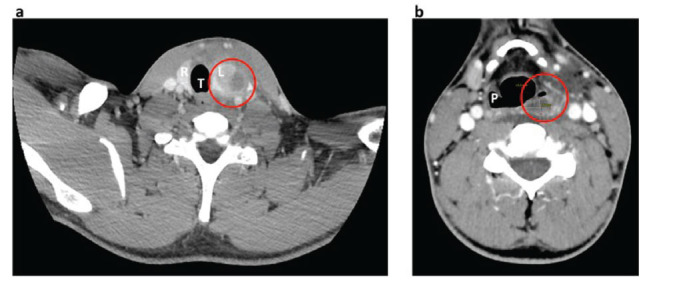
Contrast enhanced neck computed tomography. (**a**) Transversal section including thyroid. An inhomogeneous area with a large hypodense portion of colliquated appearance can be observed in red circle, involving the upper pole of the left thyroid lobe, and accompanied by significant oedema of the surrounding adipose tissue. (**b**) Transversal section at the level of the pharynx. Obliteration of the left pyriform sinus can be observed in the red circle. T=trachea, I: thyroid isthmus, L=left thyroid lobe; R= right thyroid lobe, P=pyriform sinus.

The physical examination showed swelling in the left lateral cervical area, which was extremely painful at palpation (Fig. **1**). The patient also presented with fever (body temperature 38.7°C) and heart rate at the upper limit of normality (92 bpm), with normal blood pressure (120/80 bpm) and blood oxygen saturation (SpO_2_ 98% in room air). Ultrasound examination of the neck revealed the presence of an inhomogeneous hypoechoic area in the left cervical region, measuring around 30 mm of maximum anteroposterior diameter (AP), 30 mm of transversal diameter (TS) and 45 mm of longitudinal diameter (LL), with poorly defined margins and not cleavable from the left thyroid lobe (Fig. **2**). Furthermore, ultrasound showed several enlarged lymph nodes with an elongated shape and normal hilum, suggesting an inflammatory condition. A contrast-enhanced neck computed tomography confirmed the presence of inhomogeneous area measuring 30x40x60 mm (APxTSxLL) indissociable from the upper pole of the left thyroid lobe, with peripheral contrast impregnation, presenting a large hypodense portion of colliquated appearance (Fig. **3**). This area extended to both the retropharyngeal space and the ipsilateral parapharyngeal space, flattening the left pyriform sinus and a significant oedema of latero-cervical adipose tissue surrounded it.

**Fig. (4):**
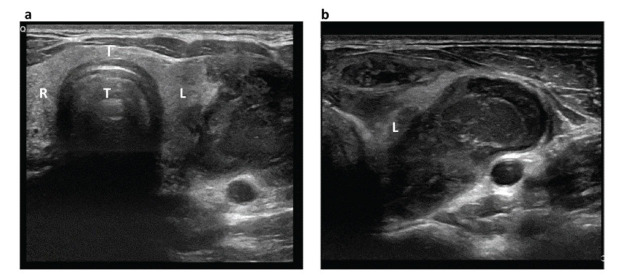
Thyroid ultrasound after one week of antibiotic treatment, in transversal section (**a**), with focus on the left lobe (**b**). An inhomogeneous area of colliquate appearance can be observed in the left thyroid lobe. T=trachea, I: thyroid isthmus, L=left thyroid lobe; R= right thyroid lobe.

Blood tests at admission showed neutrophilic leucocytosis (white blood cells 14.640/mm3, n.v 4000-11000, and neutrophils 11600/mm3, n.v. 2000-7500), along with increased inflammatory markers (ESR 19 mm/h, n.v. < 10; CRP 53.9 mg/l, n.v. <5). Moreover, the thyroid function assessment revealed suppressed TSH (TSH <0.01 mIU/L), increased free thyroxine (FT4) (20 pmol/l, n.v. 9.1-15.5), and free triiodothyronine (FT3) at the upper limit of normality (4.4 pmol/l, n.v. 2.9-4.9), suggestive for thyrotoxicosis. Antibodies anti-thyroperoxidase, anti-thyroglobulin, and anti-TSH receptor were within the normal range and the patient had no family history of thyroid disease. Therefore, AST was diagnosed and empirical antibiotic treatment was started (ceftriaxone 2 gr once a day and clindamycin 600 mg twice a day). After one week of antibiotic treatment, despite a progressive improvement of the patient’s symptoms and thyroid function, neck ultrasound documented substantial stability of the inflammatory area of 30x30x60 mm (APxTSxLL). Furthermore, the region exhibited an inhomogeneous and more markedly hypoechoic echogenicity with a colliquated appearance (Fig. **4**). The patient was therefore referred for percutaneous drainage of the abscess. The bacteria pathogen detected through culture was *G. morbillorum*.

Since *G. morbillorum* is known as an opportunistic pathogen, screening for HIV infection and immunoglobulin deficiency was conducted, but no significant findings were observed.

**Fig. (5):**
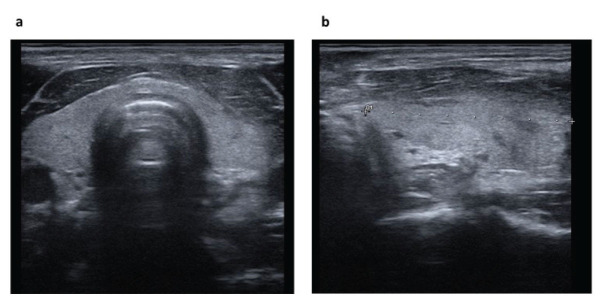
Thyroid ultrasound 3 months after discharge, both in transversal (**a**) and longitudinal section of the left thyroid lobe (**b**). A restitutio in integrum of ultrasound thyroid characteristic was observed. In particular, only a slight inhomogeneity in the left thyroid lobe was observed.

A few days after percutaneous drainage of the abscess, the patients experienced a rapid clinical improvement. Normal thyroid function was completely restored (TSH 1.0 mU/l, FT4 and FT3 within the normal range) within 2 weeks from diagnosis and the patient was therefore discharged. After 3 months, the patient still exhibited normal thyroid function (TSH 1.9 mU/L, normal FT4) and the thyroid ultrasound confirmed a globally homogeneous thyroid gland with normal vascularization, presenting only a slight inhomogeneity of the left thyroid lobe (Fig. **5**). The timeline of thyroid function and inflammatory markers is described in (Table **1**).

## 3 DISCUSSION

To the best of our knowledge, this is the first documented case of acute suppurative thyroiditis caused by *G. morbillorum*. The diagnosis of AST is supported by the early development of a large painful swelling in the anterior cervical region along with fever and clinical thyrotoxicosis. However, AST is an exceptionally rare condition, especially in the absence of predisposing factors such as immunodeficiency or neck fistulas. Thus, before diagnosing AST, it is essential to exclude more common forms of thyrotoxicosis, such as autoimmune thyrotoxicosis (Hashitoxicosis or Graves’ disease) and viral thyrotoxicosis (De Quervain's thyroiditis) [14]. Assessment of anti-thyroid antibodies (anti-thyroperoxidase, anti-thyroglobulin, and anti-TSH receptor) is helpful in identifying autoimmune thyroid diseases. In turn, the differential diagnosis between AST and De Quervain's thyroiditis (DQT) may be subtle15, but certain clinical, biochemical, and imaging features may be helpful in this regard [1, 15]. Acute suppurative thyroiditis typically exhibits a more severe initial clinical presentation compared to DQT, as well as higher fever and worse general conditions. Neutrophilic leucocytosis is primarily present in AST, while subacute thyroiditis shows relative lymphocytosis due to its viral origin. Moreover, neck ultrasound, particularly in more advanced stages, may clearly identify the presence of an abscess in AST, while DQT is distinguished by a more widespread hypoecogenicity of the glandular tissue in the absence of distinct colliquated areas.

In addition, AST usually has a shorter duration of illness (7-12 days) compared to DQT, and it does not show any response to glucocorticoid treatment. In contrast, AST has a positive response to antibiotic treatment, which has no impact on DQT.

In our patient, the overall clinical picture (presence of an abscess associated with neutrophilic leucocytosis) is oriented more towards a bacterial infection rather than a viral form. Moreover, the patient experienced rapid clinical improvement after starting antibiotic treatment and drainage of the abscess, confirming our diagnosis of AST.

The current case is also of particular interest, as *G. morbillorum* was the sole microorganism isolated within the bacterial culture. This is a gram-positive coccus, facultatively anaerobic, that represents a component of the normal flora of the oropharynx, gastrointestinal tract, and female gynaecological tract in humans [16]. There are a few reported cases of infections by *G. morbillorum* in different body districts, but never in the thyroid area. Furthermore, most of affected patients were immunocompromised, or had other risk factors for infections, such as implantable devices. However, cases of *G. morbillorum* infection in young healthy patients have also been reported [17-19].

In our case, the patient had been in good health up until the onset of AST. Furthermore, he did not have any history of known injuries in the laterocervical region, he did not have any implantable devices, and he did not have any tracheal-oesophageal fistulas, which pointed to the possibility of a spontaneous thyroid infection beginning in the oropharynx. Fortunately, an early intervention led to a full recovery, which in turn resulted in a complete restoration of normal thyroid function and ultrasound pattern.

## CONCLUSION

This is, to our knowledge, the first case report of AST caused by *Gemella morbillorum*. Infections by *G. Morbillorum* are unusual and frequently manifest in individuals with immunodeficiency or risk factors. Nevertheless, cases of infections are also reported in healthy subjects, as was the case with our patients. Interestingly, AST by *G. Morbillorum* occurred in the absence of major risk factors for thyroid infections, such as immune deficiency or neck fistulas. Due to the rapid onset of AST and its potentially life-threatening complications, prompt differential diagnosis with other forms of thyroiditis is crucial to start correct treatment and prevent serious sequelae.

## Figures and Tables

**Table 1:** Timeline of thyroid function and inflammatory markers variations during AST treatment.

**Time**	**Day 0 (start of antibiotic treatment)**	**Day 3**	**Day 9 (Percutaneous drainage of the abscess)**	**Day 14**	**Day 90**
**TSH n.v. 0.5-4.3**	<0.01	-	-	1	1.9
**FT4 (pmol/L) n.v. 9.1-15.5**	20	16.5	-	10.4	12.3
**FT3 (pmol/L) n.v. 2.9–4.9**	4.4	3.9	-	2.7	-
**ESR (mm/h) n.v < 10**	19	-	-	12	-
**CRP (mg/L) n.v < 5**	53.9	14.0	15.2	7.36	2.16

**Abbreviations:** TSH= thyroid stimulating hormone; FT4: free thyroxine; FT3 = free triiodothyronine; ESR= erythrocyte sedimentation rate; CRP= C-reactive protein.

## Data Availability

The data supporting the findings of the article are available within the article.

## LIST OF ABBREVIATIONS

AST: Acute Suppurative Thyroiditis
TS: Transversal Diameter
AP: Anteroposterior Diameter
LL: Longitudinal Diameter
DQT: De Quervain's Thyroiditis

## STANDARDS OF REPORTING

CARE guidelines and methodologies have been followed.

## References

[1] Lafontaine N., Learoyd D., Farrell S., Wong R. (2021). Suppurative thyroiditis: Systematic review and clinical guidance.. Clin. Endocrinol. (Oxf.).

[2] Desai A.K., Bonura E.M. (2021). Multi-valvular infective endocarditis from *Gemella morbillorum*.. BMJ Case Rep..

[3] Cao X., Yuan L. (2023). *Gemella morbillorum* infective endocarditis: A case report and literature review.. Open Life Sci..

[4] Yamakawa H., Hayashi M., Tanaka K., Kuwano K. (2015). Empyema due to *Gemella morbillorum* is diagnosed by 16S ribosomal RNA gene sequencing and a phylogenetic tree analysis: A case report and literature review.. Intern. Med..

[5] Valipour A., Koller H., Setinek U., Burghuber O.C. (2005). Pleural empyema associated with Gemella morbillorum: Report of a case and review of the literature.. Scand. J. Infect. Dis..

[6] Saad E., Faris M.E., Abdalla M.S., Prasai P., Ali E., Stake J. (2023). A rare pathogen of bones and joints: A systematic review of osteoarticular infections caused by *Gemella morbillorum*.. J. Clin. Med. Res..

[7] Desmottes M.C., Brehier Q., Bertolini E., Monteiro I., Terreaux W. (2018). Septic arthritis of the knee due to *Gemella morbillorum*.. Int. J. Rheum. Dis..

[8] Chotai S., Hong-joo M., Joo-han K., Jong-hyun K., Hung-seob C., Youn-kwan P., Taek-hyun K. (2010). Brain abscess caused by *Gemella morbillorum* : A case report and review of literature.. Turk Neurosurg..

[9] Abu-Heija A.A., Ajam M., Veltman J. (2018). *Gemella morbillorum* cryptogenic brain abscess: A case report and literature review.. Cureus.

[10] Villegas E., Valldeoriola F., de Otero J., Ferrer L., Oms B., Vila L., Lozano P. (2008). Meningitis by *Gemella morbillorum* with associated pituitary apoplexy: A case report.. Eur. J. Intern. Med..

[11] Hsu C.Y., Su Y.C., Wang T.L., Chong C.F., Chen C.C. (2007). *Gemella morbillorum* liver abscess.. Scand. J. Infect. Dis..

[12] Borro P., Sumberaz A., Testino G. (2014). Pyogenic liver abscess caused by *Gemella morbillorum* .. Colomb. Med. (Cali).

[13] Nagashima T., Hirata D., Yamamoto H., Okazaki H., Minota S. (2001). Antineutrophil cytoplasmic autoantibody specific for proteinase 3 in a patient with shunt nephritis induced by *Gemella morbillorum* .. Am. J. Kidney Dis..

[14] Majety P., Hennessey J.V., Feingold K.R., Anawalt B., Blackman H.R., Boyce A., Chrousos G., Corpas E., de Herder W.W., Dhatariya K., Dungan K., Hofland J. (2000). Acute and subacute, and Riedel’s thyroiditis.. Endotext.

[15] Stasiak M., Lewiński A. (2021). New aspects in the pathogenesis and management of subacute thyroiditis.. Rev. Endocr. Metab. Disord..

[16] Goldstein E.J.C., Merriam C.V., Claros M.C., Citron D.M. (2022). Comparative susceptibility of *Gemella morbillorum* to 13 antimicrobial agents.. Anaerobe.

[17] Kumar G., Al Ali A.S., Gulzar Bhatti N. (2017). Rare bacteria infecting the heart and affecting the kidney of a young child.. Case Rep. Nephrol. Dial..

[18] Boto L.R., Calado C., Vieira M., Camilo C., Abecasis F., Campos A.R., Correia M. (2011). Subdural empyema due to *Gemella morbillorum* as a complication of acute sinusitis.. Acta Med. Port..

[19] Roche M., Smyth E. (2005). A case of septic arthritis due to infection with *Gemella morbillorum* .. J. Infect..

